# Prevalence and Prognostic Implications of Vitamin D Deficiency in Chronic Kidney Disease

**DOI:** 10.1155/2015/868961

**Published:** 2015-03-26

**Authors:** Yoshitsugu Obi, Takayuki Hamano, Yoshitaka Isaka

**Affiliations:** ^1^Department of Geriatric Medicine & Nephrology, Osaka University Graduate School of Medicine, 2-2 Yamada-oka, Suita, Osaka 565-0871, Japan; ^2^Department of Comprehensive Kidney Disease Research, Osaka University Graduate School of Medicine, 2-2 Yamada-oka, Suita, Osaka 565-0871, Japan

## Abstract

Vitamin D is an important nutrient involved in bone mineral metabolism, and vitamin D status is reflected by serum total 25-hydroxyvitamin D (25[OH]D) concentrations. Vitamin D deficiency is highly prevalent in patients with chronic kidney disease (CKD), and nutritional vitamin D supplementation decreases elevated parathyroid hormone concentrations in subgroups of these patients. Furthermore, vitamin D is supposed to have pleiotropic effects on various diseases such as cardiovascular diseases, malignancies, infectious diseases, diabetes, and autoimmune diseases. Indeed, there is cumulative evidence showing the associations of low vitamin D with the development and progression of CKD, cardiovascular complication, and high mortality. Recently, genetic polymorphisms in vitamin D-binding protein have received great attention because they largely affect bioavailable 25(OH)D concentrations. This finding suggests that the serum total 25(OH)D concentrations would not be comparable among different gene polymorphisms and thus may be inappropriate as an index of vitamin D status. This finding may refute the conventional definition of vitamin D status based solely on serum total 25(OH)D concentrations.

## 1. Introduction

Vitamin D is a fat soluble secosteroid that interacts with a specific nuclear receptor similar to other steroid hormones and plays a central role in calcium and phosphate homeostasis and musculoskeletal health [1]. The past decade has brought an increasing awareness of the effects of vitamin D on several other organ systems. Ecological and observational studies have demonstrated that vitamin D deficiency, defined as a low serum total 25-hydroxyvitamin D (25[OH]D) concentration, is associated with increased risks of death and diseases such as various cardiovascular diseases, malignancies, infectious diseases, diabetes, autoimmune diseases, and kidney diseases [2]. However, more than a billion people worldwide are thought to have vitamin D deficiency or insufficiency.

Chronic kidney disease (CKD) has been identified as a risk factor for vitamin D deficiency. Indeed, the prevalence of vitamin D deficiency or insufficiency is high among patients with CKD, especially patients with end-stage renal disease and kidney transplant recipients [3, 4]. Similar to the general population, vitamin D deficiency in these patients is associated with elevated concentrations of parathyroid hormone and bone turnover markers as well as low bone mineral density [5–7]. Accumulating evidence indicates the associations of vitamin D deficiency with morbidities and mortality in patients with CKD.

In this review, we summarize the previous findings in the epidemiological and interventional studies of vitamin D in patients with CKD. The importance and implications of a genetic polymorphism of vitamin D-binding protein, which potentially overturn the current concept of vitamin D status, are also discussed.

## 2. Vitamin D Metabolism

**Figure 1:**
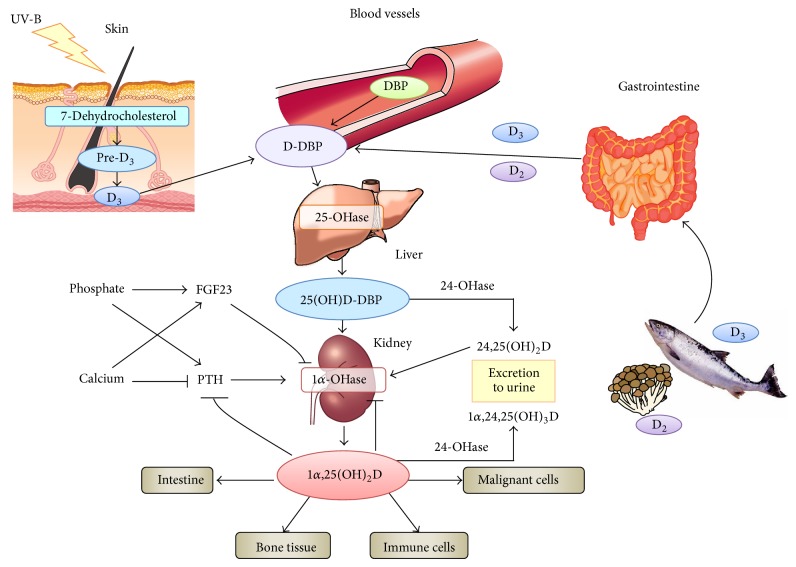
Vitamin D metabolism. DBP: vitamin D-binding protein; PTH: parathyroid hormone; FGF23: fibroblast growth factor 23.

Vitamin D is synthesized via skin exposure to ultraviolet irradiation or oral intake and binds to vitamin D-binding protein (DBP) in the blood. After being converted into 25(OH)D by 25-hydroxylase CYP2R1 of the liver in a relatively unregulated manner, it forms stable 25(OH)D-DBP complex which has a long circulating half-life of 480 hours (Figure 1, Table 1). This complex is excreted into urine and reabsorbed via megalin at renal proximal tubule cells and then converted to the active form of vitamin D, 1,25-hydroxyvitamin D (1,25(OH)_2_D), by 1*α*-hydroxylase CYP27B1. Compared to 25(OH)D, 1,25(OH)_2_D has a shorter half-life because of its lower affinity for DBP, and 1*α*-hydroxylase is also regulated by various factors including parathyroid hormone (PTH) and fibroblast growth factor-23 (FGF23). Accordingly, serum total 25(OH)D concentration, rather than the 1,25(OH)_2_D concentration, is considered as an indicator of vitamin D sufficiency.

Conventionally, 25(OH)D has been considered merely a precursor before activation; however, serum total 25(OH)D concentration is also suggested to have clinical significance. First, 25(OH)D has a binding capacity, albeit weak, for vitamin D receptors (VDR), and has hundred times higher blood concentration than 1,25(OH)_2_D (Table 1). Additionally, various extrarenal cells express megalin and cubilin as well as 1*α*-hydroxylase [8, 9]. Thus, 25(OH)D is taken up into these cells via megalin and cubilin and exerts autocrine or paracrine effects after being locally converted into the active form 1,25(OH)_2_D [10, 11]. Indeed, a number of studies have reported elevated PTH concentrations, low bone mineral density, high bone turnover, and high prevalence of fracture in population with low serum 25(OH)D concentrations even if serum calcium and 1,25(OH)_2_D concentrations were within the normal range [12, 13].

It should be noted here that extrarenal 1*α*-hydroxylase is regulated in different ways from that in renal tubular cells. For example, contrary to its effect in renal proximal tubular cells, FGF23 increases 1*α*-hydroxylase expression in parathyroid cells [14]. Also, PTH and calcium do not affect the expression and activity of 1*α*-hydroxylase in osteoblasts while interleukin-1*β*, a NF-*κ*B activator, increases its expression [15].

## 3. Vitamin D Status in CKD

FGF23 secretion from bone cells is enhanced at a relatively early stage of CKD to compensate for the phosphorus retention associated with a decrease in the number of nephrons. It inhibits the renal expression of 1*α*-hydroxylase resulting in the decrease in serum 1,25(OH)_2_D concentrations, followed by secondary hyperparathyroidism. Several studies demonstrated that FGF23 concentrations actually increase before PTH concentrations [16, 17]. Meanwhile, serum total 25(OH)D concentrations did not decrease until stage 5 CKD, while vitamin D deficiency was highly prevalent over all.

The risk factors of vitamin D deficiency include severely impaired renal function, hypoalbuminemia, urine protein/urine albumin concentrations, and diabetes [18–22]. Patient's nutritional status and inflammation influence the production of DBP in the liver, similar to that of albumin. 25(OH)D-DBP complex is excreted into urine in patients with overt proteinuria, and tubular damage in diabetic patients with CKD decreases megalin expression in the epithelial cells and thus causes decreased reabsorption of urinary 25(OH)D-DBP complex [23]. Actually, the increase in serum total 25(OH)D concentrations by nutritional vitamin D supplementation increases is diminished in patients with vitamin D deficiency and overt proteinuria [24].

## 4. Current Clinical Practice Guidelines for Vitamin D Status in Patients with CKD

According to the Kidney Disease: Improving Global Outcomes (K/DIGO) guidelines, although there is only limited evidence, nutritional vitamin D supplementation is suggested when predialysis patients with CKD were vitamin D deficient or insufficient, adjusting serum total 25(OH)D to the recommended concentration for the general population [25]. Nevertheless, the definition of insufficiency and deficiency varies among researchers and organizations. The Institute of Medicine proposed 20 ng/mL or more of serum total 25(OH)D as an indicator of sufficiency [26], while many other views support the conventional concentration of 30 ng/mL as stated in the 2003 Kidney Disease Outcomes Quality Initiative (KDOQI) guidelines [27]. However, the largest problem is that neither value has been sufficiently evaluated with respect to the association with clinical outcomes in patients with CKD. For end-stage renal disease patients on dialysis, calcitriol and active vitamin D analogues are recommended to manage secondary hyperparathyroidism if necessary, but there is no clear statement regarding optimal serum total 25(OH)D concentration or nutritional vitamin D supplementation.

## 5. Association between the Vitamin D Status and Clinical Outcomes in CKD

As mentioned above, vitamin D is a fat soluble secosteroid that interacts with a specific nuclear receptor VDR and exerts nonclassical effects on various target organs. Nevertheless, studies have consistently shown that vitamin D deficiency is highly prevalent among patients with CKD and strongly associated with various clinical outcomes. For example, serum total 25(OH)D concentrations showed significant relationships to renal outcomes such as doubling of serum creatinine or ESRD, independent of conventional risk factors in predialysis patients with CKD [28–30]. Serum total 25(OH)D concentrations were also associated with anemia and muscle weakness independent of 1,25(OH)_2_D [31, 32]. Furthermore, among patients with wide range of renal dysfunctions including ESRD, vitamin D deficiency showed the associations with vascular calcification, vascular endothelial function, cardiovascular events, and cardiovascular mortality [33–37]. A meta-analysis of these observational studies revealed an inverse association between all-cause mortality and serum total 25(OH)D concentrations [38].

In a prospective observational study of renal transplant recipients, Obi et al. identified serum total 25(OH)D concentrations as an independent predictor of annual allograft function decline [39]. Vitamin D deficiency also showed a similar relationship to the incidence of allograft rejection using intravenous methylprednisolone administration as an index. These associations were more pronounced in patients with shorter intervals after transplantation but not observed at ≥10 years after transplantation. Bienaimé et al. also reported that patients with lower serum total 25(OH)D concentrations at 3 months after transplantation exhibited lower kidney allograft function at 1 year after transplantation and had higher risk of the progression of interstitial fibrosis and tubular atrophy [40]. No association with allograft rejection was found in this study, but other cohort studies showed significant associations with cellular rejection [41, 42], consistent with the findings by Obi et al.

Those nonclassical associations of vitamin D found in observational studies have been supported by a large body of basic biological science. Supposed mechanisms of the organ-protective effects of vitamin D are as follows: the inhibition of the renin-angiotensin system and NF-*κ*B pathway [43], direct upregulation of the nitric oxide synthase transcription in vascular endothelial cells [44], and the activation of the antioxidative Keap1-Nrf2 pathway [45]. The association of vitamin D with allograft rejection in renal transplant recipients described above might be explained by the induction of regulatory T cells and the inhibition of activated T cells and B cells [36].

## 6. Interaction between the Vitamin D Status and FGF23 in Clinical Outcomes of CKD

There is a rapidly increasing number of evidences demonstrating that FGF23 is associated with mortality, the progression of CKD, and the development of cardiovascular events in patients with CKD [46]. This phosphaturic hormone secreted by bones regulates 1*α*-hydroxylase and thus confounds with calcium, phosphorus, PTH, 25(OH)D, and 1,25(OH)_2_D. Nevertheless, few studies have evaluated all of these factors simultaneously. Nakano et al. examined each independent association using a prospective cohort study of predialysis patients with CKD and identified serum concentrations of total 25(OH)D and intact FGF23, but not 1,25(OH)_2_D, as significant predictors of renal composite outcome of doubling serum creatinine and ESRD [17]. Furthermore, Hamano et al. found a nonlinear association between serum total 25(OH)D concentrations and annual kidney function decline in the same cohort; FGF23 showed a linear negative association at <23 ng/mL of total 25(OH)D while it leveled off above that concentration [47]. Meanwhile, although both low serum total 25(OH)D and high intact FGF23 concentrations were associated with cardiovascular events including heart failure in the univariate analysis, only intact FGF23 remained significant in a multivariate analysis [48]. Similarly, in an Italian study of general elderly individuals, people with higher FGF-23 concentrations and lower serum 25(OH)D concentrations had greater left ventricular hypertrophy and higher mortality while high FGF-23 concentrations but not low serum total 25(OH)D concentrations were independently associated with mortality after adjustment for other risk factors [49].

However, the extent to which FGF23 increases along with the progression of CKD could be affected by vitamin D status. The Renal Risk in Derby (RRID) study in the United Kingdom reported that intact FGF23 increased prior to PTH in patients with vitamin D sufficiency (≥20 ng/mL of serum total 25[OH]D), consistent with the previous findings [50]. On the other hand, PTH concentrations increased earlier and were relatively higher than FGF23 in patients with <20 ng/mL of serum total 25(OH)D. These results could be explained by the inhibitory effect of vitamin D against parathyroid gland and the mutual compensation of PTH and FGF23 for their phosphaturic effects. Based on these results, vitamin D status would become more important than before if FGF23 measurement becomes clinically available because FGF23 concentrations should be interpreted with caution taking both kidney function and serum total 25(OH)D concentrations into account. Further studies are still necessary to make these complicated biomarkers practically useful in clinical settings.

## 7. Nutritional Vitamin D Supplementation and CKD

Nutritional vitamin D (cholecalciferol and ergocalciferol) is widely available as an over-the-counter supplement in most countries. Notably, nutritional vitamin D is unlikely to induce hypercalcemia unless given continuously at a high dose because its 1*α*-hydroxylase-mediated activation process is regulated by many factors such as PTH, FGF23, and 24-hydroxylase (Figure 1). This is in contrast to the effects of calcitriol and active vitamin D analogues that directly increase calcium absorption and reabsorption in the intestine and kidney tubular cells, respectively. A total serum 25(OH)D concentration of <100 mg/mL is generally considered safe [1], and the United States Institute of Medicine has suggested a tolerable upper oral intake level of 4,000 IU/day [26]. Another advantage of nutritional vitamin D is that it forms a complex with DBP after being converted into 25(OH)D, which yields a long half-life (480 hours, Table 1). These characteristics allow the prescription of nutritional vitamin D at doses equivalent to weeks or months.

Patients with CKD are considered at a low risk for nutritional vitamin D-induced hypercalcemia CKD because of elevated FGF23 concentrations and tubular cell injuries. Indeed, several studies, including randomized controlled trials, a systematic review, and a metabolic balance study, have shown that although nutritional vitamin D supplementation does not significantly affect serum calcium and phosphate concentrations, it increases serum 1,25(OH)_2_D concentrations and decreases plasma PTH concentrations [51–54]. Furthermore, most studies have reported no effects of nutritional vitamin D supplementation on the concentrations of FGF23 [55–59]. Given the supposed pleiotropic effects, the safety profile, and the low cost of nutritional vitamin D, the prevalence of vitamin D supplementation has rapidly increased in the United States during the past decade (from 10% in 2003 to 44% in 2011) [60]. This may explain the reason why vitamin D status was not associated with long-term clinical outcomes including cardiovascular disease, ESRD, and death in the HOST (Homocysteinemia in Kidney and End Stage Renal Disease) study [61].

Interestingly, a randomized controlled trial showed that cholecalciferol prevented hospitalization for bone fractures or falls in ESRD patients on hemodialysis [59]. This result was consistent with those in the previous nutritional vitamin D trials that involved elderly subjects [62, 63]. These effects of vitamin D could be explained by the recent findings that skeletal muscle expresses VDR and that ligand-dependent 25-hydroxyvitamin D3 uptake is regulated and modulated by vitamin D in the primary myofibers [64]. In fact, nutritional vitamin D supplementation enhances muscle strength, especially in elderly individuals and those with vitamin D deficiency [65].

Besides its effects on musculoskeletal health, there is little concrete evidence regarding the pleiotropic effects of nutritional vitamin D. Observational studies have shown promising results such as improved endothelial function and reduced urinary albumin and TGF-*β* concentrations in predialysis patients [57, 66, 67], as well as decreased inflammatory cytokine concentrations and a reduced requirement for the erythropoiesis-stimulating agents used to manage anemia in patients on dialysis [68–70]. A small pilot study found that high-dose cholecalciferol decreased the concentration of monocyte chemoattractant protein-1 (MCP-1) but did not affect the blood concentrations of IL-6, TNF-*α*, and IL-10 [71]. Another pilot randomized controlled trial also failed to show significant beneficial effects of nutritional vitamin D on muscle strength, exercise tolerability, and health-related quality-of-life [72]. However, well-designed randomized controlled trials with adequate power have not yet been conducted.

## 8. Genetic Polymorphisms of the Vitamin D-Binding Protein and Bioavailable 25(OH)D

It has often been noted that the results of vitamin D trials vary inconsistently in terms of not only nonclassical pleiotropic effects on ill health such as cardiovascular diseases, but also the classical treatment effect against osteoporosis in general population. Even in meta-analyses, which are considered as the highest evidence, some articles reported beneficial effects of vitamin D on musculoskeletal health whereas others did not [73–75]. This discrepancy is supposed to be due to the difference in the baseline vitamin D status, baseline risk of events, doses and dosing periods of nutritional vitamin D supplementation, and adherence among the study subjects. In addition, VDR genetic polymorphism has also been suggested as an influence [76].

Powe et al. recently reported an interesting study regarding the association between bioavailable vitamin D concentrations and genetic polymorphism of DBP [77]. Although a large part (85–90%) of 25(OH)D in the blood binds to DBP as mentioned above, previous reports have reported that DBP-bound 25(OH)D could not exert its effects on target cells [78, 79]. Based on these reports, only free (<1% of total 25[OH]D) and albumin- or lipoprotein-bound fractions (10–15% of total 25[OH]D) are considered as biologically available. In fact, an observational study showed that bioavailable 25(OH)D, rather than total 25(OH)D, was associated with serum calcium and plasma PTH concentrations in patients on hemodialysis [80]. Furthermore, unique combinations of 2 common polymorphisms at rs7041 and rs4588 polymorphisms induce amino acid changes and produce 3 different phenotypes of DBP (Gc1F, Gc1S, and Gc2) which have different binding capacities to 25(OH)D [81, 82]. Accordingly, Powe et al. examined the genotypes and serum concentrations of DBP using African-Americans and Caucasians homozygous for the DBP variant and evaluated the association between bioavailable vitamin D and PTH concentrations. The following 3 important findings should be noted.

1. Regarding DBP phenotype, the frequency of Gc1F (high binding capacity) was dominant among African-Americans, whereas that of Gc1S (low binding capacity) was high among Caucasian subjects.
2. DBP blood concentrations were lowest in subjects homozygous for Gc1F (mean 93 *μ*g/mL) and highest in subjects homozygous for Gc1S (mean 468 *μ*g/mL).
3. Compared to Caucasians, African-Americans had lower serum total 25(OH)D concentrations over all but had the similar concentrations of bioavailable 25(OH)D within the quintiles of PTH concentrations.

These findings suggest that it may be inappropriate to determine vitamin D status of each individual by serum total 25(OH)D concentrations unless we examine the DBP phenotype (i.e., affinity). It might refute the conventional definition of vitamin D status using only serum total 25(OH)D concentrations. For example, the Multiethnic Study of Atherosclerosis (MESA) reported a significant association between serum total 25(OH)D concentrations and coronary heart disease in Caucasian subjects, but no association in African-American subjects. DBP polymorphisms potentially explain this racial difference [83] as well as the previously reported poor response to nutritional vitamin D supplementation in patients with low serum total 25(OH)D concentrations [84].

An* in vitro* study has also demonstrated that monocytes cultured with lower-affinity DBPs showed more potent induction of cathelicidin by 25(OH)D or 1,25(OH)_2_D [85]. Thus, DBP genetic polymorphisms may influence the association of serum total 25(OH)D concentrations with clinical outcomes and the effect of nutritional vitamin D supplementation. Conversely, another report found that DBP concentrations did not modify the effect of cholecalciferol on PTH [86]. These results will require further verification in additional intervention studies.

## 9. Conclusion

A large body of evidence concerns the beneficial effects of vitamin D on musculoskeletal health as well as various diseases. Observational studies have also shown an association of the vitamin D status with clinical outcomes in patients with CKD. However, vitamin D status is affected by various factors, including the seasonality of measurement, physical activity, nutritional and inflammatory status, diabetes, and urinary protein excretion. This raises significant concerns that those associations except for musculoskeletal health might be the result and not the cause of the patient's condition [87]. However, given the fact that the association with mortality is stronger in observational studies in which the prevalence of nutritional supplementation was low, nutritional vitamin D might have some pleiotropic effect on health [88]. Nonetheless, observational studies tend to overestimate true causal relationships.

Additionally, DBP gene polymorphism might be of particular importance in future human vitamin D studies. For example, Japanese studies have reported prevalence rates of 67%, 20%, and 13%, for the DBP polymorphisms Gc1F, Gc1S, and Gc2, respectively, suggesting heterogeneity of this gene among Asian populations [89–91]. Although the importance of vitamin D in CKD is well acknowledged, further observational and interventional studies of various races are still needed to evaluate the clinical associations of vitamin D and its related genes and to test the clinical efficacy of nutritional vitamin D supplementation.

## Figures and Tables

**Table 1:** The difference in characteristics of 25-hydroxyvitamin D and 1,25-dihydroxyvitamin D.

	Affinity to vitamin D receptor	Serum total concentration	Half-life	Risk of hypercalcemia
25(OH)D	(1)^∗^	9.0–34.0 ng/mL (500)^∗^	480 hrs	Low

1,25(OH)_2_D	(100–200)^∗^	20–60 pg/mL (1)^∗^	15 hrs	High

^∗^Relative value.
